# Vaccines: Preventing Disease Protecting Health

**DOI:** 10.3201/eid1011.040728

**Published:** 2004-11

**Authors:** Marc A. Strassburg

**Affiliations:** * Los Angeles County, Department of Health Services, Los Angeles, California, USA

**Keywords:** vaccine, disease prevention

Vaccines: Preventing Disease Protecting Health does not provide the type of vaccine-specific information as Plotkin and Orenstein's Vaccines (*1*), nor does it provide the details on the immune system of Bloom and Lambert's The Vaccine Book (*2*). The book does not cover every important vaccine issue, such as ethical issues in vaccine trials and the conduct of clinical trials; most critically, it lacks an index. But these limitations are minor compared to what the book provides.

This relatively small book provides state-of-the-art information by those who are directly involved with vaccine and immunization programs. The book evolved from a meeting held November 25–27, 2002, in Washington, D.C., at which many of the world's top vaccine scientists reported on their research. As the book jacket states, the roster of authors reads like a Who's Who in vaccine research and public health immunization programs. This publication comes from the Pan American Health Organization (PAHO), the World Health Organization (WHO) Regional Office, which has been at the forefront of almost every major vaccine initiative for the past 30 years, including the eradication of polio, elimination of measles, and strategies to control rubella and neonatal tetanus. These programs have served as models emulated by other WHO regions in the world. The editor, Ciro A. de Quadros, former director of PAHO's Vaccine and Immunization Program, has a scholarly hand, as well as an eye towards what is practical and useful. This book conveys not only what has been achieved in the arena of vaccine-preventable diseases in the 30 years since the first such conference was convened by PAHO in 1970 but also what is most likely to happen during the next 30 years.

The chapters are quick and painless reading, in many cases directly from those involved in the research described. For example, Peter F. Wright, one of the leading researchers on respiratory syncytial virus (RSV) vaccines, provides this candid assessment: "The road leading to the development of a vaccine for the prevention of RSV has been so difficult and the prospects for a vaccine remain so daunting, that only the impact of the disease provides the imperative for researchers to continue in their quest." Michiaki Takahashi, one of the developers of the varicella vaccine, provides insights on breakthrough cases occurring in "15–20% of vaccine recipients." Takahashi also reports on the status of a vaccine to prevent herpes zoster in older persons. Roger Glass et al. provide a succinct update on rotavirus vaccines currently in development and human trials.

Even highly technical aspects of new vaccine development, such as DNA vaccines, are dealt with clarity in relatively brief presentations. Topics not always found in other vaccine books include those on anti-hookworm vaccine, mucosal vaccines, and oral vaccines derived from transgenic plants. The chapter on new polio vaccines is coauthored by Eckard Wimmer, who achieved worldwide fame by using the poliovirus' known genetic sequence to synthesize that virus from the building blocks of DNA and a broth of other chemicals.

Overall, the book includes several scenarios. "The Present" section concerns controlling diseases for which there are available vaccines; "The Cutting Edge" section concerns recently introduced vaccines. "The Future" concerns candidate vaccines on the horizon; "The Quest" section concerns challenging areas for vaccine development, which include HIV/AIDS, malaria, and dengue. Other sections include "New Concepts," "Delivery Systems," "Bioterorrism," "Regulatory and Safety Issues," as well as "Health Financing." To some degree, the book's descriptive and detailed table of contents makes up for the absence of an index. While hard to believe, this book about vaccines and vaccine programs is hard to put down because of its readability.
